# *Lightella neohaematopini*: A new lineage of highly reduced endosymbionts coevolving with chipmunk lice of the genus *Neohaematopinus*

**DOI:** 10.3389/fmicb.2022.900312

**Published:** 2022-08-01

**Authors:** Jana Říhová, Kayce C. Bell, Eva Nováková, Václav Hypša

**Affiliations:** ^1^Department of Parasitology, Faculty of Science, University of South Bohemia, České Budějovice, Czechia; ^2^Department of Mammalogy, Natural History Museum of Los Angeles County, Los Angeles, CA, United States; ^3^Department of Biology, Museum of Southwestern Biology, University of New Mexico, Albuquerque, NM, United States; ^4^Department of Zoology, Denver Museum of Nature and Science, Denver, CO, United States; ^5^Institute of Parasitology, Biology Centre, ASCR, v.v.i., České Budějovice, Czechia

**Keywords:** genome evolution, insect symbionts, lice, symbiosis, coevolution, *Neohaematopinus pacificus*

## Abstract

Sucking lice (Anoplura) are known to have established symbiotic associations multiple times with different groups of bacteria as diverse as Enterobacteriales, Legionellales, and Neisseriales. This diversity, together with absence of a common coevolving symbiont (such as *Buchnera*, in aphids), indicates that sucking lice underwent a series of symbiont acquisitions, losses, and replacements. To better understand evolution and significance of louse symbionts, genomic and phylogenetic data are needed from a broader taxonomic diversity of lice and their symbiotic bacteria. In this study, we extend the known spectrum of the louse symbionts with a new lineage associated with *Neohaematopinus pacificus*, a louse species that commonly parasitizes North American chipmunks. The recent coevolutionary analysis showed that rather than a single species, these lice form a cluster of unique phylogenetic lineages specific to separate chipmunk species (or group of closely related species). Using metagenomic assemblies, we show that the lice harbor a bacterium which mirrors their phylogeny and displays traits typical for obligate mutualists. Phylogenetic analyses place this bacterium within Enterobacteriaceae on a long branch related to another louse symbiont, “*Candidatus* Puchtella pedicinophila.” We propose for this symbiotic lineage the name “*Candidatus* Lightella neohaematopini.” Based on the reconstruction of metabolic pathways, we suggest that like other louse symbionts, *L. neohaematopini* provides its host with at least some B vitamins. In addition, several samples harbored another symbiotic bacterium phylogenetically affiliated with the Neisseriales-related symbionts described previously from the lice *Polyplax serrata* and *Hoplopleura acanthopus*. Characterizing these bacteria further extend the known diversity of the symbiotic associations in lice and show unique complexity and dynamics of the system.

## Introduction

Establishment of an obligate mutualistic symbiosis with nutrient-providing bacteria is an important evolutionary event that allows some insect groups to exploit new ecological niches (Sudakaran et al., 2017). Due to mutual dependence, such host-symbiont systems often undergo long-term coevolution manifested by mutually mirrored phylogenies between the host and the symbiont (Moran et al., 1993; Chen et al., 1999; Sauer et al., 2000; Dhami et al., 2013). This type of essential nutritional mutualist is usually called a primary symbiont (P-symbiont) characterized by several typical features, such as genome reduction, low GC content, and genomic deterioration of metabolic capacities (Toft and Andersson, 2010). In several insect groups, particularly those feeding on plant xylem or phloem sap, coevolution of the symbiont with the host can reach hundreds of millions of years into the past (Moran et al., 1993; Spaulding and von Dohlen, 1998; Sauer et al., 2000). In contrast, secondary symbionts (S-symbionts) are symbiotic bacteria with diverse phenotypes, not essential for their hosts (Stouthamer et al., 1999; Zchori-Fein and Perlman, 2004; Moran et al., 2005; Toh et al., 2006; Nováková et al., 2009), which are usually acquired more recently and may accompany P-symbionts in a particular taxon, or even a population of the host (McCutcheon et al., 2019).

In sucking lice of the order Anoplura, several obligate symbiotic bacteria have been described, generally thought to provide the hosts with compounds missing in their diets (Hypsa and Krizek, 2007; Fukatsu et al., 2009; Boyd et al., 2014, 2016; Allen et al., 2016; Rihova et al., 2021). Although all extant Anoplura share similar lifestyles and diets, no common ancient P-symbiont has been maintained through all lineages. Instead, several groups of sucking lice have acquired their symbionts independently from bacterial taxa as diverse as Enterobacteriales (Fukatsu et al., 2009; Boyd et al., 2014, 2016), Legionellales (Rihova et al., 2017), and Neisseriales (Rihova et al., 2021). While from the deep phylogenetic perspective the symbionts of sucking lice seem to form a diverse assemblage of different bacteria (Allen et al., 2016), at the more recent phylogenetic level some symbiotic lineages show tendencies to coevolve with their host for a limited period, and display features common for P-symbionts. These are, for example, the *Puchtella* lineages described from closely related species of *Pedicinus* (Boyd et al., 2017) and the *Legionella polyplacis* known at least from two *Polyplax* host species, *P. serrata* and *P. spinulosa* (Hypsa and Krizek, 2007). Some other louse symbionts seem to have been acquired more recently, and their genomes appear to have undergone a transition from S-symbionts toward P-symbionts (Boyd et al., 2016; Rihova et al., 2021). This ambiguous mode of symbiosis, i.e., short periods of coevolution intermixed with frequent losses and acquisitions, raises a question on the significance of the symbionts for their louse hosts. While considered obligate mutualists, essential for the host survival and reproduction, the exact role of the louse symbionts is not clear. Seventy-year-old experimental evidence obtained for *Pediculus humanus* suggests that this louse may depend on provisioning of most B vitamins by its symbiont (Puchta, 1955). However, genomic comparison of the louse symbionts for which complete genomes are available shows considerable inconsistency in preservation of their metabolic pathways, including those for B vitamins (Rihova et al., 2021). To better understand the evolution and significance of louse-symbiont systems, much broader taxonomic diversity has to be examined using genomic and phylogenomic tools.

In this study, we extend the current list of the investigated louse-symbiont systems by taking advantage of the recently described codiversification between several chipmunk species (Rodentia: Sciuridae: *Tamias*) and their lice of the genus *Neohaematopinus* (Bell et al., 2021). These lice are primarily parasites of sciurid rodents in Asia and North and Central America, where one species, *N. pacificus*, was described from western North American chipmunks in the subgenus *Neotamias* (Bell et al., 2015, 2021). Coevolutionary analysis between the chipmunks and lice indicates that rather than a single polyxenic species, the lice classified within *N. pacificus* form a cluster of separated phylogenetic lineages each specific to a single chipmunk species or group of closely related species (Bell et al., 2021). Utilizing metagenomic data and phylogenetic background from this coevolutionary study, we identify a new putatively obligate and mutualistic symbiont of lice, show its coevolution with the louse hosts, and reconstruct its metabolic capacity related to its likely symbiotic role.

## Materials and methods

### Screening and assembly of chipmunk lice metagenomic data

Metagenomic data from 21 specimens of *Neohaematopinus* lice was downloaded from the NCBI SRA database (Sequence Read Archive; Leinonen et al., 2011; Table 1). Initially, we employed a comprehensive search for bacteria by classifying metagenomic reads from each sample using the GOTTCHA2-v.2.1.7 signature-based metagenomic taxonomic profiling tool (Freitas et al., 2015) implemented in KBase (Arkin et al., 2018) with database built from NCBI RefSeq Release 90. The full taxonomic output reports were summarized at the strain and genus level in R with package phyloseq (McMurdie and Holmes, 2013). All the bacterial strains with relative abundances above 0.1% in any sample were considered.

**TABLE 1:** Overview of metagenomic binning and taxonomy assignment.

SRR data code	Number of paired reads	Read length (nt)	Bin count	GTDB classified MAG bins	Assigned taxonomy: family; genus	Bin total length (nt) and contig count
SRR5088469	154952566	100	18	bin.004.fa	Enterobacteriaceae	458733 (5)
				bin.006.fa	Burkholderiaceae; *Variovorax*	1723338 (455)
				bin.009.fa	Burkholderiaceae; *Burkholderia*	7009440 (797)
				bin.015.fa	Hyphomicrobiaceae; *Hyphomicrobium*	422320 (157)
				bin.016.fa	Rhizobiaceae; *Mesorhizobium*	9399685 (16661)
				bin.017.fa	Sphingomonadaceae; *Sphingomonas*	4635438 (84)
SRR12483222	69397816	160	116	bin.022.fa	Enterobacteriaceae	464809 (14)
SRR12483221	34725622	160	40	bin.015.fa	Enterobacteriaceae	459627 (5)
				bin.024.fa	Xanthobacteraceae; *Bradyrhizobium*	7557328 (558)
SRR12483220	32486852	160	121	bin.044.fa	Enterobacteriaceae	460455 (6)
SRR12483219	55847912	160	156	none	NA	NA
SRR12483218	80798234	160	105	bin.012.fa	Enterobacteriaceae; *Puchtella*	440570 (34)
SRR12483217	27859822	160	72	bin.059.fa	Lactobacillaceae; *Lactobacillus*	254633 (74)
				bin.061.fa	Enterobacteriaceae; *Puchtella*	378565 (47)
SRR12483215	23212912	160	104	none	NA	NA
SRR12483214	27929696	160	166	bin.058.fa	Enterobacteriaceae; *Wigglessworthia*	202321 (55)
SRR12483213	41335766	160	47	bin.014.fa	Lactobacillaceae; *Lactobacillus*	500811 (86)
				bin.041.fa	Xanthobacteraceae; *Bradyrhizobium*	6871847 (970)
SRR12483212	27333640	160	96	none	NA	NA
SRR12483211	21275976	160	105	none	NA	NA
SRR12483210	46206712	160	130	none	NA	NA
SRR12483209	29071184	160	136	bin.012.fa	Enterobacteriaceae; *Puchtella*	317573 (72)
SRR12483208	38144898	160	107	bin.072.fa	Enterobacteriaceae; *Puchtella*	462982 (6)
SRR12483207	27742464	160	10	bin.019.fa	Enterobacteriaceae	449562 (3)
SRR12483206	38930820	160	60	bin.014.fa	Xanthobacteraceae; *Bradyrhizobium*	2938854 (759)
				bin.043.fa	Neisseriaceae	1148191 (218)
SRR12483204	71767598	160	78	bin.015.fa	Enterobacteriaceae	262 958 (53)
SRR12483203	20092028	160	43	none	NA	NA
SRR12483202	60251848	160	118	none	NA	NA
SRR12483201	34764372	160	92	bin.087.fa	Enterobacteriaceae	465231 (6)

Assemblies of the metagenomic data were generated using SPAdes v.3.13.0 (Bankevich et al., 2012) with the option, meta. The CONCOCT v.1.1 software (Alneberg et al., 2014) implemented in Kbase (kb_concoct 1.3.4) was ran with default parameters, using Bowtie2 (Langmead and Salzberg, 2012) as a default read mapper, to organize the contigs into putative metagenome-assembled genomes (MAGs) based on their nucleotide composition and depth of coverage. Metagenome-assembled genomes were classified into bacterial taxa using GTDB-Tk (Chaumeil et al., 2020) against the Genome Taxonomy Database (GTDB) v.R06-RS202 (258,406 bacterial and archaeal genomes) based on average nucleotide identity with a reference genome, the genome’s position in the reference tree, or relative evolutionary divergence and placement of the genome in the reference. The GOTTCHA read classification and GTDB-Tk classification of MAGs (Supplementary Table 1) both indicated the presence of a prospective obligate endosymbiont in majority of the samples. In the GOTTCHA classification (Supplementary Table 1), a number of reads were assigned to the taxa *Buchnera*, *Baumannia*, and *Evansia*. GTDB-Tk classification of MAGs revealed several Enterobacteriaceae, including *Puchtella* sp. str. PRUG, endosymbiont of the louse *Pedicinus baddi* (Table 1). Among the other assignments, a potentially interesting taxon in respect to endosymbiotic interactions, was the Neisseriaceae-related bacterium (see “Results and discussion” section).

### Refining metagenome-assembled genomes and completing genome drafts

To refine Enterobacteriaceae and Neisseriaceae MAGs, i.e., to detect potentially missed contigs and to remove the incorrectly binned ones, we used the following procedure (scheme provided in Supplementary Figure 1). The Enterobacteriaceae MAGs were present in twelve louse metagenomic assemblies, in five of them by only few long contigs (Table 1). For these twelve meta-assemblies, we filtered all putative Enterobacteriaceae contigs based on their GC content and coverage. For each assembly, these values were inferred from the contigs present in its Enterobacteriaceae MAG. From these extended bins, we then selected subsets of all prokaryotic contigs based on the ORFs density as predicted in Geneious Prime v.2020.2.5 (Kearse et al., 2012). To confirm that this approach detected all contigs belonging to the Enterobacteriaceae symbiont, we performed an alternative BLAST-based screening. We prepared databases from all 21 MAGs and screened them by BLASTn search (set to default E-value 10.0 and one best hit) using all genes from *Puchtella* sp. str. PRUG (NZ_LKAS01000001.1 + NZ_LKAS01000002.1) and two close relatives *Wiglessworthia glossinidia* (BA000021.3; NC_016893.1) and *Blochmannia pensylvaticus* str. BPEN (NC_007292.1) as queries. In addition, we used annotated genes from the best draft (the sample SRR12483207; for annotation procedure see below) as queries to screen the remaining 20 meta-assemblies by BLASTx searches (Camacho et al., 2009) set to default E-value (10.0) and one best hit. For all the contigs retrieved by these approaches we checked their taxonomical origin by BLASTn search (set to default E-value 10.0 and three best hits) against the nucleotide (nt) database. The Neisseriaceae-related MAG was present in a single meta-assembly (SRR12483206) and was largely fragmented. To refine this MAG, we used the same approach as described above for the Enterobacteriaceae. To screen all other meta-assemblies for possible presence of this bacterium, we applied the BLAST-based approach with the genes of the two Neisseriaceae-related louse symbionts (CP046107 and WNLJ00000000) as queries.

For the putative taxonomic assignment of each gene in the least fragmented genome of the Enterobacteriaceae symbiont (L207) we employed BLASTx search against the nr database set to three best hits (Supplementary Table 2). The completeness of the L207 draft was assessed by BUSCO v.4 (Simao et al., 2015) and for the Enterobacteriaceae symbiont also by the size convergence (see “Results and discussion” section). All genome drafts were annotated by PROKKA (Seemann, 2014) and RAST (Aziz et al., 2008) and deposited in GenBank under the BioProject accession number PRJNA813538 for the twelve most complete assemblies of Enterobacteriaceae symbiont (L222, L469, L220, L207, L201, L221, L218, L208, L2017, L209, L214, and L204) and the two most complete assemblies of Neisseriaceae-related symbiont (N206 and N204). To detect candidate pseudogenes in the annotated L207 and N206 assemblies, we used bioinformatic tool Pseudofinder (Syberg-Olsen et al., 2021).

## Phylogenetic analyses

### Phylogenetic position of the new Enterobacteriaceae symbiont

Because preliminary analyses suggested phylogenetic position of the dominant symbiont within Enterobacteriales, we downloaded a representative set of available proteomes for each major lineage of Enterobacteriales from NCBI and JGI databases (proteome accession numbers provided in Supplementary Table 3A) including the putatively closest relatives indicated by the binning process and BLASTp against NCBI database (*Puchtella* and *Wigglessworthia*). Several gammaproteobacteria representing other orders were used as outgroups: *Vibrio cholerae* O1 biovar eltor str. N16961 (Vibrionales), *Pseudomonas aeruginosa* PAO1 (Pseudomonadales), *Candidatus* Evansia muelleri CEM1.1 (Oceanospirillales), and *Xanthomonas citri* pv. *vignicola* strain CFBP7111 (Xanthomonadales). BLASTp searches with default parameters (E-value 10.0) and *Salmonella enterica* (accession NZ_CP065718) protein queries for 14 orthologs from Husnik et al. (2011) were used to retrieve sufficiently long and reliably aligned sequences (list of the orthologs provided in Supplementary Table 3B) from all the proteomes. For the new Enterobacteriaceae symbiont, we included two of the least fragmented assemblies (L207 and L221), for which we identified the corresponding orthologs by BLASTp (with default E-value 10.0) and verified these by visual inspection and by the gene’s annotations. The single-gene amino-acid matrices were aligned by MAFFT v.7.450 (Katoh et al., 2002) using the E-INS-i setting implemented in Geneious Prime v.2020.2.5 (Kearse et al., 2012), visually inspected, and the genes concatenated. Ambiguously aligned regions were removed by Gblocks (Castresana, 2000) using the less stringent option. The concatenated matrix (“phylogenetic matrix”) was analyzed using maximum likelihood (ML) and Bayesian inference (BI) for the phylogenetic reconstruction. The resulting matrix consisted of 7,438 amino acids. The ML tree was inferred using PhyML v.3.3 (Guindon et al., 2009) implemented in Geneious Prime with 100 bootstrap replicates and run under the best fitting CpREV + G + I evolutionary model selected by Akaike criterion (AIC) by smart model selection (SMS) algorithm (Lefort et al., 2017) implemented in the PhyML v.3.0 web server (Guindon et al., 2010). The BI analyses were performed using two different approaches. First, MrBayes v.3.2.6 (Ronquist et al., 2012) was run with four chains for 2,000,000 generations under the CpREV + G + I model. The chain convergence was evaluated in Tracer v.1.6 (Rambaut et al., 2018) and by the standard deviation of split (<0.01) and PSRF+ (reached the value 1.0). Since the *Neohaematopinus* symbionts formed a very long branch, placed within a cluster of other long-branched symbiotic bacteria, we also used PhyloBayes MPI v.1.8 (Lartillot et al., 2013) with CAT-GTR model to minimize possible artifacts due to the aberrant character of the sequences. The analysis was run for 50,000 generations and the quality of convergence was evaluated by the bpcom and tracecomp assessments. The list of bipartition differences indicated that while for most bipartitions the parameter dropped below 0.1, for two bipartitions it remained above 0.3. These problematic bipartitions included taxa distant from our focus, particularly the species of *Erwinia*. To improve the convergence and verify correctness of the topology, we prepared a “reduced phylogenetic matrix” containing 68 taxa (designated in Supplementary Table 3A) and retained the matrix of 7,438 amino acids. This matrix was analyzed in PhyloBayes with the same parameters (CAT-GTR, 50,000 generations), resulting in a drop of the maxdiff parameter to 0.057.

### Host and the Enterobacteriaceae symbiont coevolution

To assess congruence between the host and the symbiont, we prepared host and Enterobacteriaceae symbiont matrices restricted to the datasets which provided sufficient data for the symbiont (12 host/symbiont samples sharing five orthologs; Supplementary Table 3C). Due to different characters and sizes of the matrices, the host and symbiont phylogenies were computed by different methods. The host phylogeny was reconstructed by two different methods. The first method employed ML analysis of 1,107 concatenated nuclear loci from Bell et al. (2021). The reconstruction was performed in IQ-TREE v2.0 (Minh et al., 2020) with the best model (GTR + F + R10) determined by ModelFinder (Kalyaanamoorthy et al., 2017) and support assessed by 1,000 ultrafast bootstrap replicates (Hoang et al., 2018), as implemented in IQ-TREE. In the second approach, we used IQ-TREE v2.0 to generate individual gene trees for the 1,107 nuclear loci and based on these trees, we then estimated a species tree in ASTRALIII v5.7.3 (Zhang et al., 2018), with local posterior probabilities (Sayyari and Mirarab, 2016). For the assemblies of the 12 symbionts, we concatenated five shared protein single-copy orthologs (COGs) determined by Orthofinder (Emms and Kelly, 2019) to build a “coevolutionary matrix” (list of used COGs provided in Supplementary Table 3D). The sequences were aligned in MAFFT v.7.450 with E-INS-i settings and processed by Gblocks using the less stringent options. The resulting matrix consisted of 1,150 amino acids. For the phylogenetic reconstructions we used ML and BI methods. The ML tree was reconstructed with 100 bootstrap replicates using the web-based PhyML with best fitting model determined by AIC using SMS function of the online PhyML server v.3.0 (HIVb + G + F). The BI trees were inferred using MrBayes v.3.2.7 under JTT + G + F evolutionary model selected by jModelTest2 v.2.1.10 (Darriba et al., 2012). Four chains were run for 10,000,000 generations and chain convergences were checked in Tracer v.1.6 and evaluated by standard deviation split (<0.01) and PSRF+ (reached the value 1.0). Both the host and the symbiont trees were rooted to fit the *N. pacificus* topologies published in Bell et al. (2021). To evaluate the possible effect of gene number on the symbiont phylogeny (number of single-gene orthologs shared by all samples shown in Supplementary Table 3C), we also analyzed by ML, a taxonomically reduced matrix (11 symbiont samples), containing fifty shared protein single-copy orthologs (Supplementary Table 3D) determined by OrthoFinder. The sequences were aligned in MAFFT v.7.450 with E-INS-i settings and processed by Gblocks using the less stringent options. The resulting alignment included 6,379 amino acids. The ML tree was reconstructed with 100 bootstrap replicates using the web-based PhyML with the best fitting model determined by AIC using SMS function in the online PhyML server v.3.0 (Q.bird + G + F).

### Phylogenetic position of the Neisseriaceae-related symbiont

To determine the phylogenetic position of the bacterium binned using CONCOCT and classified as a member of Neisseriaceae, we prepared a dataset of Neisseriales proteomes available from the NCBI database, including the putatively closest relatives indicated by BLASTp against NCBI database (Neisseriaceae-related symbionts of lice). We used representatives from other bacterial orders as outgroups, two betaproteobacteria from the order Burkholderiales, *Acidovorax* sp. KKS102 and *Burkholderia cepacia*, one alphaproteobacterium *Rhizobium leguminosarum*, and one gammaproteobacterium, *Legionella pneumophila* subsp. *pneumophila* str. Philadelphia 1. Accession numbers of the proteomes are provided in Supplementary Table 3E. For the Neisseriaceae-related symbionts, we included sequences from the two most complete assemblies (N206 and N204). A concatenated amino acid matrix (“Neisseriales matrix”) was constructed for 30 single-gene orthologs (COGs) identified by OrthoFinder v.2.4.0 and processed in the same manner as described above for Enterobacteriaceae symbiont matrices (list of COGs provided in Supplementary Table 3F). The resulting matrix consisted of 4,465 amino acids. Phylogenetic reconstructions were performed by ML and BI methods. We used PhyML for ML reconstruction using 100 bootstrap replicates and the LG + G + I + F evolutionary model as determined by AIC using SMS algorithm in the online PhyML server v.3.0. The selected LG + G + I + F evolutionary model was also used for BI tree reconstruction in MrBayes v.3.2.7. The BI analysis was run in four chains for 10,000,000 generations and the convergences of the chains were checked in Tracer v.1.6 and evaluated by standard deviation split (<0.01) and PSRF+ (reached the value 1.0).

## Genomes and metabolic pathways comparison

To compare the genome structure and reveal the possible rearrangements, we performed two synteny analyses. The first comparison included concatenated contigs representing five genome drafts of the new Enterobacteriaceae symbiont. The samples were selected to maximize two parameters, coverage of the symbiont’s phylogenetic diversity and high completeness of the genome draft. Since several contigs contained only a single rRNA gene, and their position could not be reliably assessed, they were not included in the set. The second analysis compared these five genomes with the closest relative, i.e., *Puchtella* sp. str. PRUG (NZ_LKAS01000001.1 + NZ_LKAS01000002.1). The analyses were carried out using two programs, Mauve (Darling et al., 2010) and Clinker (Gilchrist and Chooi, 2020). Average nucleotide identity (ANI) of the genomes included in synteny analyses was calculated using a web-based ANI calculator (Yoon et al., 2017). Accession numbers for the corresponding genomes are provided in Supplementary Table 3G.

Assessment of metabolic capacities was based on the most complete and least fragmented genome drafts of the Enterobacteriaceae (L207) and Neisseriaceae (N206) symbionts. The Enzyme Commission (EC) numbers from the PROKKA output and the KEGG orthologs (KO) assigned by BlastKOALA (Kanehisa et al., 2016) were used to map the metabolic pathways in KEGG Mapper (last accessed: November 2021).^1^ For the pathways assumed to potentially play a role in the symbiosis, absence of the genes identified as missing was verified using BLAST search. The metabolic reconstructions of B vitamins for the symbionts were compared to the metabolic pathways of several other louse symbionts with available genomes in NCBI, i.e., *Puchtella* sp. str. PRUG (NZ_LKAS01000001.1 + NZ_LKAS01000002.1), *Riesia pediculicola* USDA (GCF_000093065.1), *Legionella polyplacis* (GCA_002776555.1), *Sodalis*-like endosymbiont of *Proechinophthirus fluctus* (GCA_001602625.1), Neisseriaceae bacterium PsAf (GCA_017114885.1), and Neisseriaceae bacterium HaMa (GCA_016864895.1). The KO numbers for compared genomes are deposited in Mendeley Data under the doi link https://doi.org/10.17632/cks86467mv.6.

### Proposal for the genus and species name of Enterobacteriaceae symbiont

As the results of our phylogenetic analyses show that Enterobacteriaceae symbionts form a new monophyletic lineage within the Enterobacteriaceae family, we propose the name for all included strains as “*Candidatus* Lightella neohaematopini,” gen. nov., sp. nov. (hereafter, *Lightella neohaematopini* for a simple reference). The genus name *Lightella* refers to evolutionary biologist Jessica E. Light, who devoted part of her scientific career to phthirapteran research, and the species name *neohaematopini* refers to the genus of its insect host, *Neohaematopinus pacificus*.

## Results and discussion

### Taxonomic profiling of metagenomic reads and metagenomic bins

Initial rapid taxonomic assignment of metagenomic reads provided the first insight into the bacterial content of 21 SRA datasets. On average, only 0.4% reads from each dataset were assigned to Bacteria detecting altogether 359 unique bacterial strains (Supplementary Table 1). At the genus level, the highest proportion of bacterial reads were consistently classified to *Cutibacterium*, *Lactobacillus*, *Staphylococcus, and Bradyrhizobium*. In some of the samples, i.e., SRR12483201, SRR12483218, and SRR12483221, a number of bacterial reads were assigned to known symbiotic taxa, i.e., *Buchnera*, *Baumannia*, and *Evansia* (Supplementary Figure 2). While the omnipresent taxa (*Cutibacterium*, *Lactobacillus*, *Staphylococcus*, *and Bradyrhizobium*) likely represent common human, environment or extraction kit related contamination typical for low biomass samples (Salter et al., 2014), identification of reads classified as obligate insect symbionts point out the association with similarly AT-rich bacteria.

The metagenomic assemblies reflected different qualities, sequencing depth and length of 21 SRA datasets (Table 1). The binning process resulted in a wide range in the number of bins (18–166) generated from each of the assemblies. Only 21 bins from 14 datasets were taxonomically assigned to Bacteria. The low number of bacterial bins (among 1,920 retrieved), further referred as MAGs, can be explained by the default settings used for the binning process (including min. contig length of 1,000 nt) and/or by the metagenomic origin of the SRA datasets containing high proportions of eukaryotic contigs. In contrast to the metagenomic taxonomic profiling of raw reads suggesting the omnipresence of *Cutibacterium*, *Lactobacillus*, *Staphylococcus*, *and Bradyrhizobium* genera, we have only retrieved MAGs for *Bradyrhizobium* and *Lactobacillus* from four of the meta-assemblies. It is difficult to determine the causes of this discrepancy, but it could be due to contamination with fragmented DNA, biased amplification during library preparation, or simply assignment inaccuracies stemming from the classifiers and databases we used. The majority of MAGs (12) assembled from twelve samples were identified as Enterobacteriaceae. While for seven of those the taxonomy was only resolved to the family level, five bins were assigned to symbiotic genera, i.e., *Puchtella* and *Wigglessworthia*. In addition, a single MAG representing another potentially symbiotic associate was identified as the family Neisseriaceae in a single dataset. With respect to the focus of this study, the two bacterial groups (Enterobacteriaceae and Neisseriaceae) were of particular interest and were included in further analyses.

### *Lightella neohaematopini*: A new lineage of Enterobacteriaceae symbiont

Using BLASTx and BLASTn searches, the sequences of the new enterobacterial lineage were detected in all except one sample (SRR12483202), but the quality of these genome drafts (i.e., number of contigs and their overall lengths) varied among the SRA datasets (Table 2). The size comparisons indicate that the best drafts represent almost complete genomes, as with the growing quality of the assemblies, the drafts converge to the same size (Table 2). For eight assemblies, the size of the drafts ranged between 463,996 and 461,635 bp, with the longest contigs in the five best assemblies exceeding 217 kb. While this convergence suggested that the drafts reached a high level of completeness, BUSCO produced very low values. Depending on the database, completeness for sample L207 was estimated as 45.7, 41.3, and 45.2% for Proteobacteria, Gammaproteobacteria, and Enterobacteriales, respectively (Supplementary Table 4). Such low values, however, are common even for complete genomes of some symbiotic bacteria (*Candidatus* Riesia pediculischaeffi PTSU: 59.5%; *Sulcia muelleri* strain GWSS: 22.8%; values taken from the UniProt database).^2^ The least fragmented assembly L207 (SRA sample SRR12483207) contained five contigs. This remaining fragmentation seems to be caused partly by the presence of two copies of the 23S rRNA gene, which were chimerically assembled in a single contig and could not be separated using this data (Supplementary Figure 3). This fact is also reflected by the presence of a short contig containing only the 23S rRNA gene, with coverage almost twice as high as that in the other contigs. Summarizing these parameters, the five least fragmented and most complete drafts (Table 2) were considered almost complete genomes and were used in the subsequent comparative analyses. The overview provided in the Table 3 shows that this bacterium (represented by the L207 genome assembly) possesses features similar to the closest assigned taxon, *Puchtella* sp. str. PRUG (also see below for the phylogenetic analyses and Supplementary Figure 4D for 16S rRNA gene similarities), e.g., genome size, GC content, coding density, number of protein coding sequences, absence of transposases, mobile elements or phage-relates sequences. In contrast to this similarity and phylogenetic relatedness, virtually no synteny was detected between the *L. neohaematopini* (L207) and *Puchtella* sp. str. PRUG genomes. The dotplot produced by MUMMER v.3 (Kurtz et al., 2004), shown in Supplementary Figure 4, indicates that during their diversification from a common ancestor, one or both lineages underwent substantial genome rearrangements.

**TABLE 2:** Lengths of symbiotic contigs extracted from *Neohaematopinus* lice meta-assemblies.

*Neohaematopinus pacificus* sample name	SSR data code	*Lightella neohaematopini draft genome code*	Number of *Lightella neohaematopini* contigs	Total length of *Lightella neohaematopini* contigs (bp)	Length of the longest *Lightella neohaematopini* contig	Neisseriaceae* symbiont draft genome Code	Number of Neisseriaceae* symbiont Contigs	Total length of Neisseriaceae* symbiont contigs (bp)	Length of the longest Neisseriaceae* Symbiont contig
DZTM1119Np	SRR12483222	L222	14	463,996	105,366	N222	3	6,579	3,880
DZTM377Np	SRR5088469	L469	7	463,572	217,438	N469	14	8,463	2,334
DZTM1701Np	SRR12483220	L220	7	463,522	188,166	N220	7	1,722	283
MVZ225305Np	SRR12483207	L207	5	462,938	217,084	N207	1	276	276
NK217036N	SRR12483201	L201	6	462,716	216,235	N201	24	13,211	5,588
DZTM1620Np	SRR12483221	L221	6	462,700	216,229	N221	7	1,765	290
DZTM2189Np	SRR12483218	L218	45	462,561	37,759	–	–	–	–
ZM.13956Np	SRR12483208	L208	6	461,635	216,026	N208	5	1,247	297
DZTM230Np	SRR12483217	L217	102	451,834	27,981	N217	5	1,314	282
ZM.13998Np	SRR12483209	L209	142	419,545	10,090	N209	6	1,484	278
DZTM2717Np	SRR12483214	L214	205	395,449	7,732	N214	6	2,034	689
MSB84515Np	SRR12483204	L204	105	347,616	11,111	N204	653	620,299	11,296
DZTM268Np	SRR12483215	L215	223	102,538	2,639	N215	30	7,499	295
DZTM708Np	SRR12483211	L211	231	92,062	1,150	N211	1	249	249
NK181766Np	SRR12483203	L203	144	51,326	1,557	–	–	–	–
DZTM203Np	SRR12483219	L219	134	39,424	660	N219	7	1,835	291
DZTM946Np	SRR12483210	L210	75	21,336	734	–	–	–	–
DZTM2776Np	SRR12483213	L213	3	12,058	1,097	N213	31	8,034	346
MVZ225310Np	SRR12483206	L206	46	11,224	3,732	N206	459	1,476,030	17,945
DZTM584Np	SRR12483212	L212	7	1,740	284	N212	4	1,058	307
NK215220Np	SRR12483202	–	–	–	–	–	–	–	–

The most complete and least fragmented genome drafts of L. neohaematopini are highlighted in green. The L. neohaematopini samples are ordered according to the total length of the concatenated genome drafts. *Neisseriaceae-related.

**TABLE 3:** Comparison of the main characteristics of the two new symbionts (the best drafts) with their closest relatives.

Genome	Louse host	Mammal host	Genome size (bp)	GC content (%)	Coding density (%)	CDS	Predicted proteins	Hypothetical proteins	Pseudogens	Transposases	Phage-related sequences	Mobile elements	BUSCO evaluation (%)
*Lightella neohaematopini* (L207)	*Neohaematopinus pacificus*	*Tamias alpinus*	462,938	22.2	90.5	449	443	23	29	0	0	0	45.2
*Puchtella* sp. str. PRUG	*Pedicinus badii*	*Procolobus rufomitratus*	558,122	24.2	92.4	602	547	34	47	0	0	0	72.7
Neisseriaceae-related symbiont (N206)	*Neohaematopinus pacificus*	*Tamias obscurus*	1,476,030	33.7	75.7	1,501	1,472	515	133	2	2	0	62.2
Neisseriaceae-related symbiont (PsAf)	*Polyplax serrata*	*Apodemus flavicollis*	1,814,374	33.7	89.4	1,739	1,660	336	106	5	0	0	85.8
Neisseriaceae-related symbiont (HaMa)	*Hoplopleura acanthopus*	*Microtus arvalis*	1,607,498	33.4	83.5	1,369	1,303	207	11	11	0	0	80.9

Annotation of the genome drafts resulted in similar sets of genes for the five best assemblies (annotations for corresponding genomes are deposited in Mendeley Data under the “doi” link https://doi.org/10.17632/cks86467mv.6). The number of protein coding genes varied between 435 and 443 (415–423 for genes with functional annotations and 19–23 for hypothetical genes). In three strains, the annotation contained two copies of 16S rRNA gene. In two strains the second copy was missing, most likely because of its position at the end of a contig (Supplementary Figure 4). Only one copy of 23S rRNA gene was found in the metagenomic assemblies and was placed on a short separated contig. Identical fragments of its sequences were, however, detected at the ends of two different contigs, suggesting that the 23S rRNA gene is also present in two copies and is one of the causes of the genome fragmentation (Supplementary Figure 3). The alignments obtained by Clinker and Mauve implemented in Geneious revealed the completely syntenic nature of the five strains (Supplementary Figure 4). Although the fragmentation into the contigs (up to seven) does not allow for an alignment in a strict sense, the complete syntenies along all contigs suggests that there has not been any rearrangement of the gene order. The few mismatches in the alignments were caused by the following: (1) Aligning inaccuracy at the ends of the contigs, (2) missing gene predictions or different annotations, and (3) real differences in absence/presence of the gene. In some cases, these causes are not easily recognized (e.g., missing annotation *vs.* absent sequence), but they account for only a small fraction of the genomic content (406 genes are present and identically annotated in all five genomes).

The BLAST-based verification of the common origin of all selected contigs produced hits of a broad taxonomic range. Although altogether the analysis supported *L. neohaematopini* as a member of Enterobacteriaceae, due to the aberrant nature of the sequences (manifested by very low GC content of 22.2% and long branches in phylogenetic trees), it failed to reveal a specific closely related bacterium. Instead, the best hits included various taxa of Enterobacteriaceae, often the highly modified symbiotic bacteria such as *Buchnera*, *Blochmannia*, *Baumannia*, *Wigglessworthia*, and *Sodalis* (BLASTx results for L207 presented in Supplementary Table 2).

### Phylogenetic position of *Lightella neohaematopini*

**FIGURE 1:**
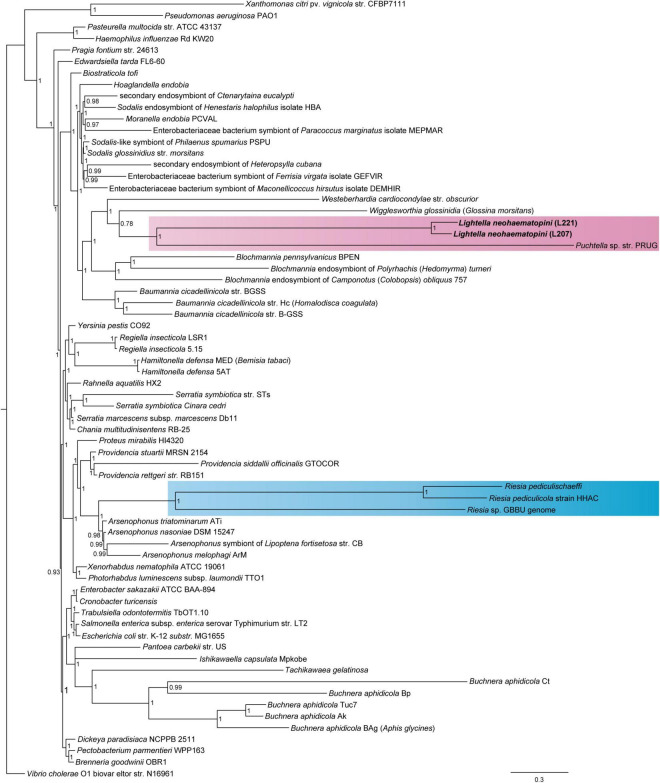
Position of *Lightella neohaematopini* within Enterobacteriales revealed by PhyloBayes analysis (BI under CAT-GTR model) of the concatenated 14-protein “reduced phylogenetic matrix” (7,438 aa). Clustering of *L. neohaemotopini* together with the *Puchtella* sp. str. PRUG is indicated by pink background. Clustering of another louse symbiont, the genus *Riesia*, within *Arsenophonus* cluster is highlighted by blue background. Values at the nodes show posterior probabilities.

Considering different qualities of the genome drafts, we performed phylogenetic analyses with two different data sets. The first data set of 14 genes (7,438 amino acids), which included two lineages of *L*. *neohaematopini* (L207, L221) and 100 (the “phylogenetic matrix”) or 63 (the “reduced phylogenetic matrix”) additional members of Enterobacteriales, produced a tree in which the two strains of *L*. *neohaematopini* formed a monophyletic cluster on a long branch, placed as a sister group to *Puchtella* (Figure 1 and Supplementary Figures 5–7). This relationship was obtained by both methods, maximum likelihood and Bayesian inference (MrBayes and PhyloBayes). However, the position of these two sister taxa (*L. neohaematopini* and *Puchtella*) as well as the arrangement of other symbiont taxa differed between the analyses. While maximum likelihood and the MrBayes analyses placed the *L. neohaematopini* + *Puchtella* lineage within a large cluster of symbionts on long branches (Supplementary Figures 6, 7), the PhyloBayes analysis split this cluster into several distinct lineages (Figure 1 and Supplementary Figure 5). In this tree, *L. neohaematopini* + *Puchtella* clustered in vicinity of *Wigglessworthia* and *Blochmannia*, two obligate insect symbionts with strongly modified genomes (i.e., highly reduced and AT-rich). Since highly aberrant sequences of symbiotic bacteria are known to be affected by long-branch attraction (Charles et al., 2001; Husnik et al., 2011), the cluster of long-branched symbionts produced by the maximum likelihood and MrBayes analyses is likely to be artificial and does not reflect real phylogenetic relationships. We therefore consider the PhyloBayes-derived topology, which is also compatible with the results obtained by Husnik et al. (2011), as a more reliable reconstruction of evolutionary history. However, with the current data on louse symbionts, the phylogenetic position of long-branched *L. neohaematopini* does not allow for interpretation of its symbiotic origin. Considering the phylogenetic distance between their hosts (Light et al., 2010), it is unlikely that *L. neohaematopini* and *Puchtella* share a common lice-associated symbiotic ancestor. We rather suggest that these bacteria established their relationship independently with different louse lineages and evolved into mutualists. This view is compatible with the broader phylogenetic picture showing that sucking lice acquired their symbionts several times from different groups of bacteria (Rihova et al., 2021). It should also be noted that no data are currently available on other *Neohaematopinus* species or related genera, such as *Haemodipsus* or *Sathrax*. If these lice possess symbionts related to *L. neohaematopini*, their inclusion in the analysis could in principle “break” the long branch of *L. neohaematopini* and allow for a more reliable phylogenetic placement, as well as evolutionary interpretation of its origin.

**FIGURE 2:**
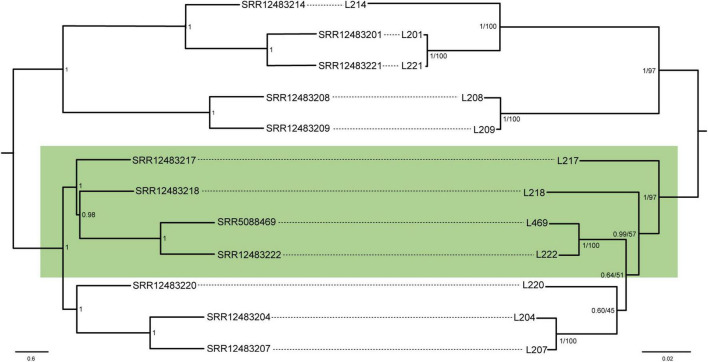
Coevolutionary reconstruction comparing phylogenetic trees of the lice and *Lightella neohaematopini.* The host tree was inferred by ASTRAL-III method from 1,107 individual gene trees, each tree derived from one of the 1,107 nucleotide matrices by IQ-TREE using GTR + F + R10 model. The symbiont tree was inferred by MrBayes (BI under JTT + G + F model) and PhyML (ML under HIVb + G + F model) from the amino-acid five-gene “coevolutionary matrix” (1,150 aa). Lengths of the branches in the symbiont tree correspond to the BI results. Values at the nodes of the symbiont tree show posterior probabilities/bootstrap. Values at the nodes of the host tree show local posterior probabilities. The green box highlights the conflicting parts of the phylogenies.

The second data set composed of 1,150 amino acids (five genes) from 12 strains of *L. neohaematopini* produced a ML tree (Supplementary Figure 8) and BI tree (Figure 2) with topologies closely corresponding to that of the host lice. Almost perfect congruence was found between the *L. neohaematopini* tree and the host tree produced by the ASTRAL-III method (Figure 2). The only discrepancy was the monophyly (louse tree) *vs.* paraphyly (symbionts) of the four-taxa cluster highlighted in Figure 2. This difference is most likely caused by an incorrect topology of one or both trees. As indicated in the symbiont tree in Figure 2, the conflicting phylogeny included two branches (indicated by green background in Figure 2) which are among the shortest in the tree and supported with low bootstrap values and posterior probabilities. Similarly, the host trees produced by two different methods (ASTRAL-III and IQ-TREE) differ in arrangement of these groups (Figure 2 and Supplementary Figure 9A). The extension of the ortholog number by reducing the set of symbiont samples to 11 (L204 removed) resulted in slightly different topology but did not produce complete louse-symbiont congruence (Supplementary Figure 9B). Despite these minor incongruences, most likely caused by phylogenetic artifacts, the overall close correspondence between the host and the symbiont tree provides strong evidence for the common origin of these symbionts and their coevolution with the host.

### Metabolic capacity and symbiotic role of *Lightella neohaematopini*

**FIGURE 3:**
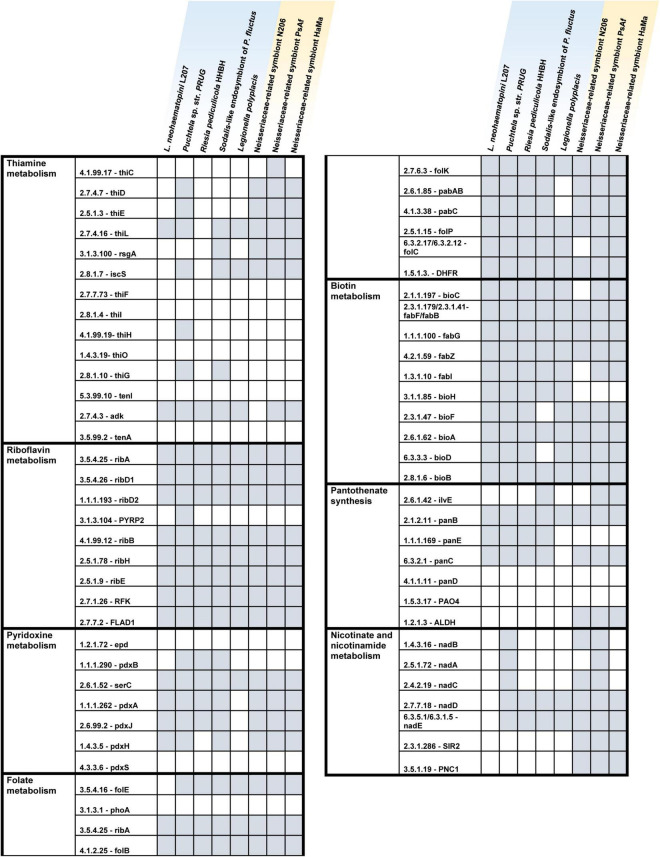
Comparison of B-vitamins pathways for *Lightella neohaematopini* L207, Neisseriaeceae-related symbiont N206 and other louse symbionts (blue background = γ-proteobacteria, yellow background = β-proteobacteria). Presence of the particular genes is indicated by gray background.

The genome of *L. neohaematopini* shows several features typical for obligate mutualists in insects (e.g., low GC content, reduced size), indicating a possible metabolic role in its louse host. In correspondence with the strong genome reduction, this new symbiont has considerably limited metabolic capacity (Figure 3 and Supplementary Figure 10). Two categories of metabolites are most often considered compounds provided by an obligate symbiont to the blood-sucking host, amino acids and B vitamins (Nogge, 1981; Hosokawa et al., 2010; Snyder et al., 2010; Rihova et al., 2017). The *Neohaematopinus*-symbiont seems to have lost capacity for synthesis of most of the amino acids, only retaining pathways for lysine, glycine, and serine (for the serine/glycine pair, a category of “cyclic pathway” was established in Rihova et al. (2021), as many symbionts have the capacity for interconverting between them but cannot synthesize these amino acids from glucose; Supplementary Figure 10). On the other hand, *L. neohaematopini* retained considerable numbers of genes involved in synthesis of several B vitamins, namely riboflavin, folate, biotin, pantothenate, and pyridoxine (Figure 3). Of these vitamins, *L. neohaematopini* contains complete pathways for biotin. This pathway is also retained by three other louse-associated symbionts included in the comparison, *Puchtella* sp., *Riesia pediculicola*, and *L. polyplacis*, suggesting that biotin might be the most important compound provided to lice by their symbionts. In the riboflavin pathway, one gene is missing (5-[amino-6-(5-phospho-D-ribitylamino)uracil phosphatase; EC: 3.1.3.104] but as shown previously (and also here in Figure 3), this gene is missing in otherwise complete pathways of many other symbiotic bacteria, and it is thus likely that its function can be replaced by some other not yet identified gene (Rihova et al., 2021). The pathways for two additional B vitamins, folate and pyridoxine, are missing two and three genes, respectively, some of them missing in all genomes included in the analysis. It is difficult to deduce from this pattern if *L. neohaematopini* can produce (or contribute to production) of these vitamins. It has been previously shown that some seemingly missing genes in these pathways playing role in mutualistic relationships can be present on a plasmid (Kirkness et al., 2010). However, this does not seem to be the case here: when using orthologs of these genes as BLAST query, we did not find their homologs in any of the 21 analyzed assemblies. For pantothenate synthesis, the symbiont’s genome contains three genes (panB, panC, and panG) while the absence of the panD is likely to be compensated by the host (Price and Wilson, 2014). These pathways also do not contain any of the genes identified as potentially being pseudogenized. Altogether, this overview indicates a role for the symbiont in provisioning several B vitamins, or at least biotin, like some other louse symbionts (Boyd et al., 2017; Rihova et al., 2017, 2021). A comparison with its close relative, *Puchtella* sp. str. PRUG (symbiont of *P. baddi* from red colobus monkey *Procolobus rufomitratus*), shows highly similar metabolic capacities of these two louse symbionts (Figure 3).

### Neisseriaceae-related symbionts

**FIGURE 4:**
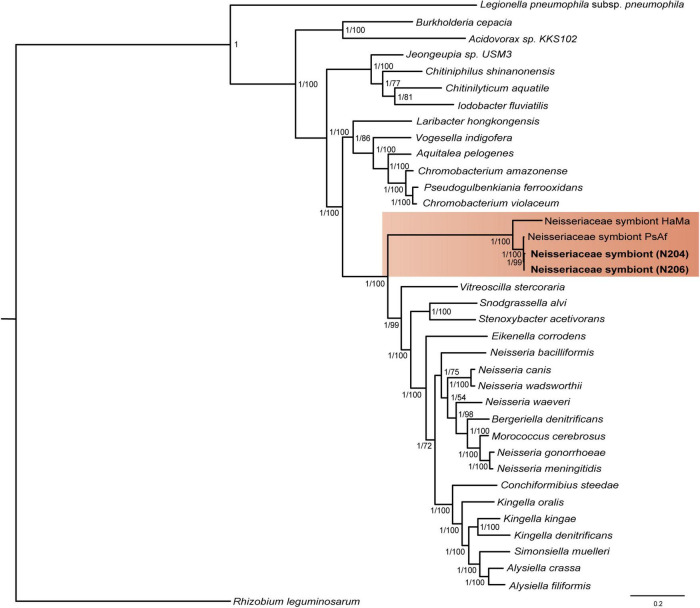
Clustering of the N206 and N204 symbionts together with the Neisseriaceae-related symbionts from Rihova et al. (2021), indicated by orange background. The tree was inferred by MrBayes (LG + G + I + F model) and PhyML (LG + G + I + F model) from the amino-acid 30-gene “Neisseriales matrix” (4,465 aa), lengths of the branches in the tree of the symbiont correspond to the BI tree results. Values at the nodes of the symbiont tree show posterior probabilities/bootstrap supports.

Apart from *L. neohaematopini*, the binning process detected a putative Neisseriales-related symbiont (Table 1). Its sequences were present in 17 assemblies, most of them in highly fragmented and incomplete form (Table 2). The most complete genome draft (N206) is 1,476,030 bp long (the longest contig reaching 17,945 bp) with a low GC content (33.7%). It contains 1,472 predicted protein coding genes, 515 of them annotated as hypothetical proteins (Table 3). The two assemblies (N206 and N204) included in the phylogenetic analysis cluster together with the Neisseriaceae-related symbionts PsAf and HaMa previously described from *Polyplax serrata* and *Hoplopleura acanthopus* lice, respectively (Rihova et al., 2021; Figure 4). The HaMa was also retrieved as the first BLAST hit when the contigs assigned to Neisseriaceae in the 17 assemblies were used as a query against the nt NCBI database. The clustering on a long common branch and high branch support (both ML and BI analyses) suggest that all these Neisseriaceae-related bacteria may have originated from a single symbiotic ancestor (Supplementary Figure 11 and Figure 4). This phylogenetic position and the features typical for bacteria undergoing adaptation to symbiosis (e.g., low GC content and high number of pseudogenes) indicate the symbiotic nature of these bacteria. The main characteristics of the N206 genome, and its comparison to the other Neisseriaceae-related symbionts are summarized in Table 3. Since *N. pacificus* harbor *L. neohaematopini* as a typical obligate mutualistic P-symbiont, the significance of the Neisseriaceae-related symbiont for the host is unclear. Furthermore, the highly fragmented and possibly incomplete genome assembly (BUSCO completeness value 62.2%) does not allow for a reliable reconstruction of its metabolic capacities (in Figure 3 and Supplementary Figure 10 we show the capacities which can be inferred from this data). It is, however, interesting to see that these bacteria are present in several louse genera, possibly playing the role of either S-symbionts (*N. pacificus, P. serrata*) or even P-symbionts (*H. acanthopus*).

The results presented in this study provide two pieces of information relevant for broader insight into the evolution of symbiosis between lice and bacteria. First, the new putative obligate mutualist, *L. neohaematopini*, extends the list of bacterial lineages which have established mutualistic symbiosis with different groups of sucking lice. This further supports the view that sucking lice underwent an exceptionally dynamic process of symbiont acquisitions and replacements. Phylogenetic match of *L. neohaematopini* with *Neohaematopinus pacificus* lice from different chipmunk species provides strong evidence that these symbionts were acquired in a single evolutionary event and have been maintained during the host’s radiation. This codiversification process likely reflects the nutritional role of these bacteria. A comparison with other known louse symbionts suggests that the compounds most likely responsible for this dependence are several B vitamins. In contrast, the second potentially symbiotic bacterium is related to Neisseriaceae-related symbionts already known from two different groups of lice (Rihova et al., 2021). Interestingly, in *Neohaematopinus pacificus* lice the co-occurrence of these two bacteria resembles the situation in *Polyplax serrata*, where the mutualistic *Legionella polyplacis* is occasionally accompanied by the Neisseriaceae-related symbiont. We expect that addition of so far unexplored louse lineages will further extend overall diversity of their symbionts, but it can also help to resolve some of the current phylogenetic and evolutionary uncertainties.

## Data availability statement

The original contributions presented in this study are included in the article/Supplementary material, further inquiries can be directed to the corresponding author.

## Author contributions

VH, EN, and JŘ conceived the study. All authors contributed to the data analyses, interpretation of the results and writing the manuscript.

## Footnotes

1
http://www.genome.jp/kegg/mapper.html

2
www.uniprot.org/proteomes
