# Supplementary material for: Lightella neohaematopini: A new lineage of highly reduced endosymbionts coevolving with chipmunk lice of the genus Neohaematopinus
Source: Front Microbiol. 2022 Aug 1;13:900312. doi: 10.3389/fmicb.2022.900312 (PMC9376444; doi:10.3389/fmicb.2022.900312)
Supplement: Supplementary file 1 [file Data_Sheet_1.PDF]

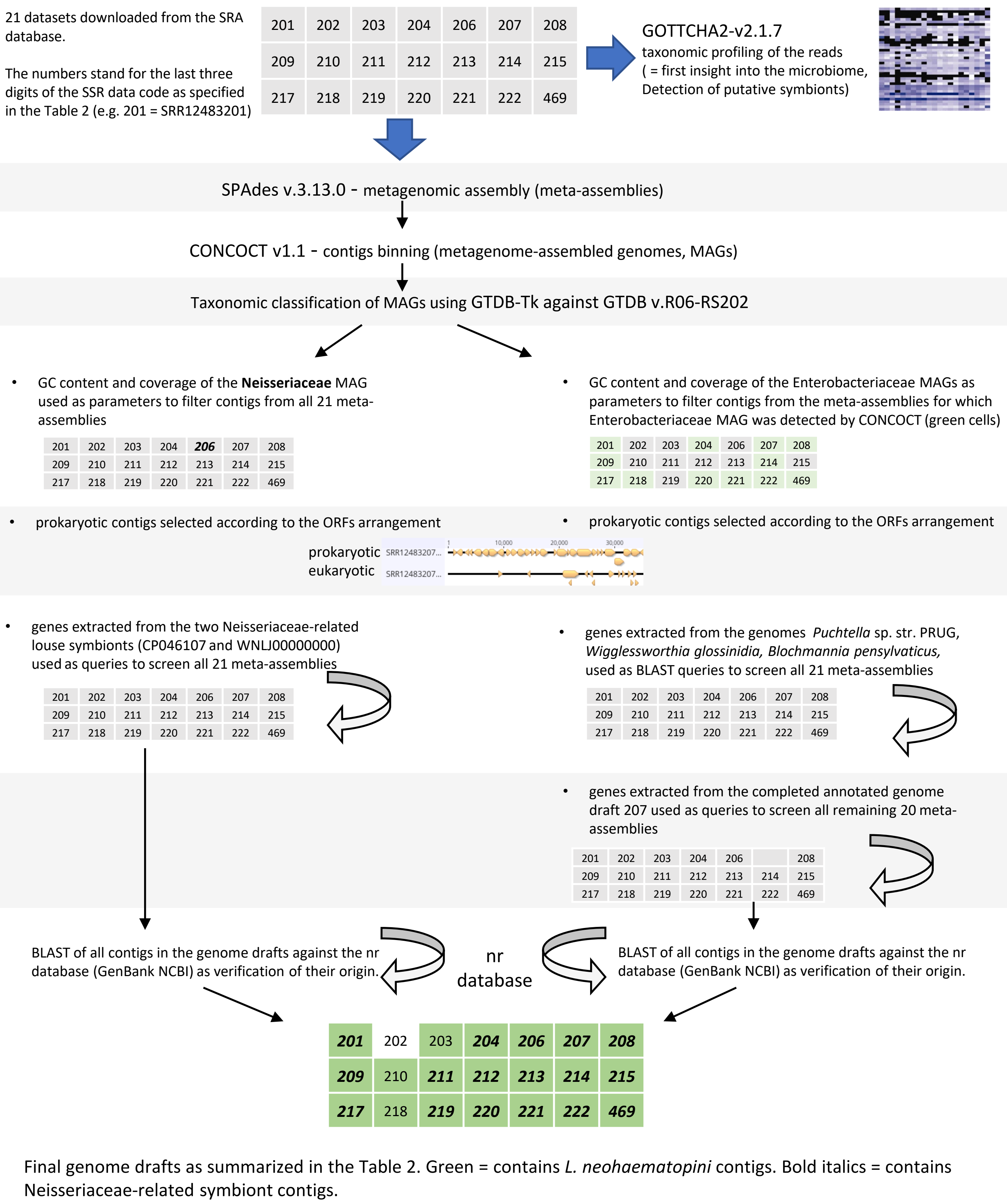

SupplementaryFigure1. Methodological scheme representing the procedure used to obtain genome drafts from the raw SRA data.

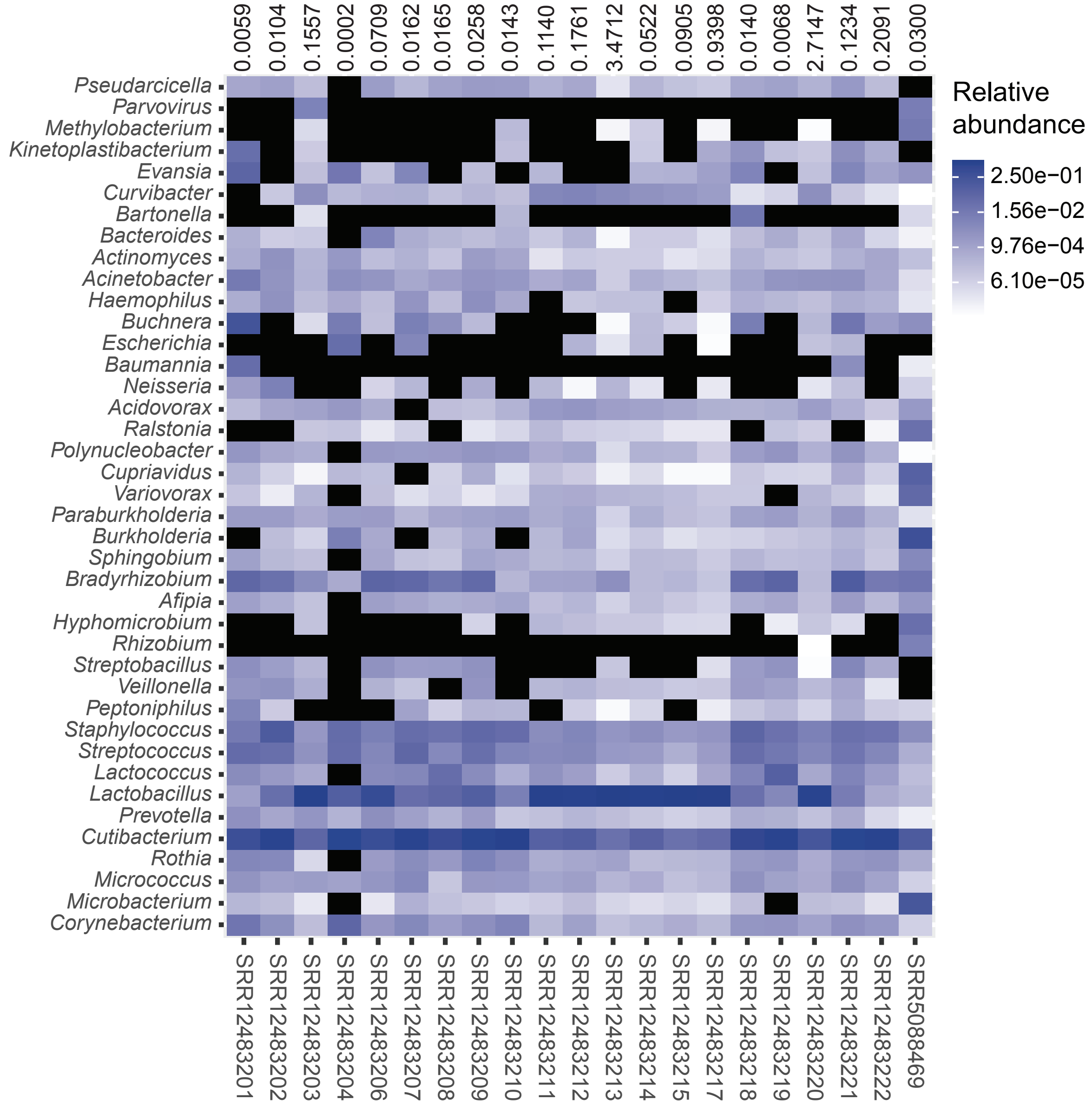

SupplementaryFigure2: Relative abundance of the first 40 most abundant bacterial genera found across the sample set of analyzed metagenomic reads based on GOTTCHA2 taxonomic assignment (see Materials and Methods). The numbers stand for proportion of reads in each SRA dataset assigned to Bacteria. Black color designates absence of the genus.

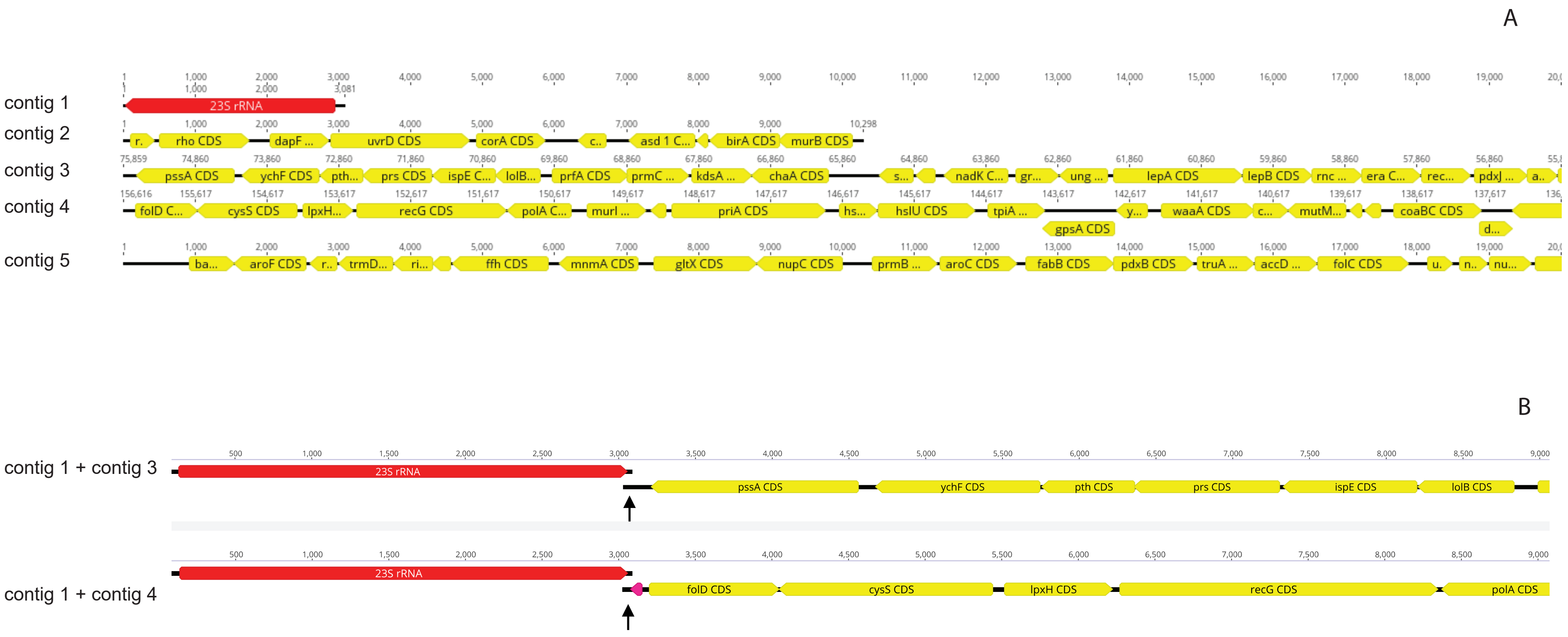

SupplementaryFigure3. A - five contigs of the L207 draft (only initial 20 kb are shown for the contigs 3 - 5). B - contig 1, containing only 23S rRNA gene, assembles with two different contigs (3 and 4), each containing different genes. The arrows point to the overlaps with 100% identity. Black line = strand of the DNA sequence, pink part = tRNA. The axis provides a scale in bp.

A

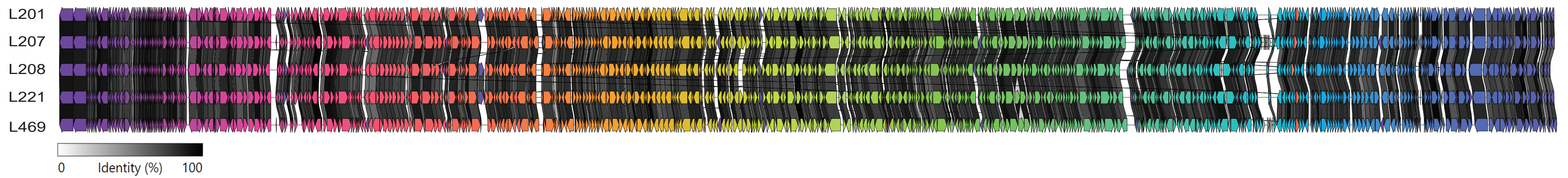

B

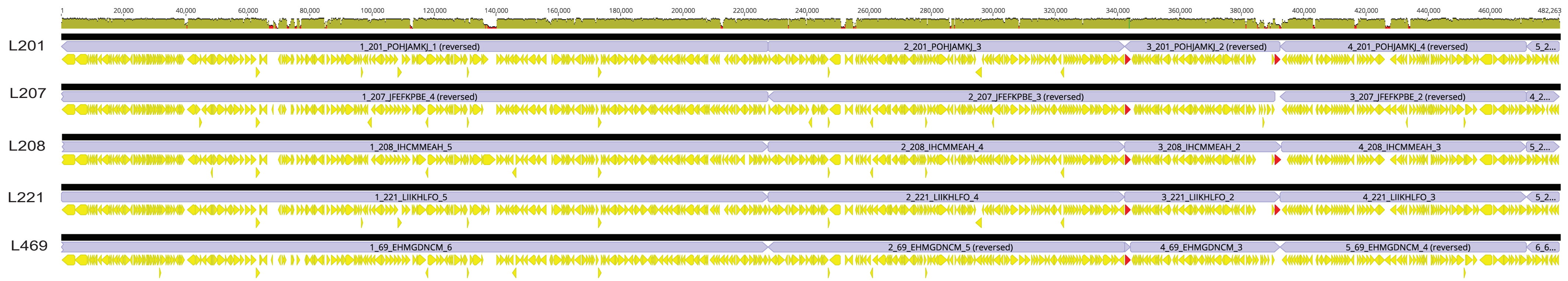

C

genome drafts

	L201	L207	L208	L221	L69
**L207**	86.49				
**L208**	85.55	86.58			
**L221**	99.88	86.5	85.59		
**L69**	86.53	92.23	86.66	86.54	
Puchtella	67.66	67.25	67.52	67.72	68.62

genome drafts	coverage (%)
L201/L208	76.28/76.45
L469/L208	72.02/72.34
L469/L201	78.60/78.78
L221/L208	76.55/76.72
L221/L201	99.51/99.51
L221/L469	78.85/78.68
L207/L208	72.57/72.73
L207/L469	67.52/67.38
L207/L201	77.53/77.53
L207/L221	77.62/77.62
Puchtella /L208	19.05/23.17
Puchtella /L201	21.12/26.63
Puchtella /L469	17.49/21.17
Puchtella /L221	20.90/25.36
Puchtella /L207	21.44/26.02

D

16S rRNA genes

	L201	L207	L208	L221	L469
**L207**	97.212				
**L208**	97.654	97.524			
**L221**	100	97.212	97.654		
**L469**	97.524	98.215	97.903	97.524	
Puchtella	86.725	87.217	86.888	86.725	87.327

E

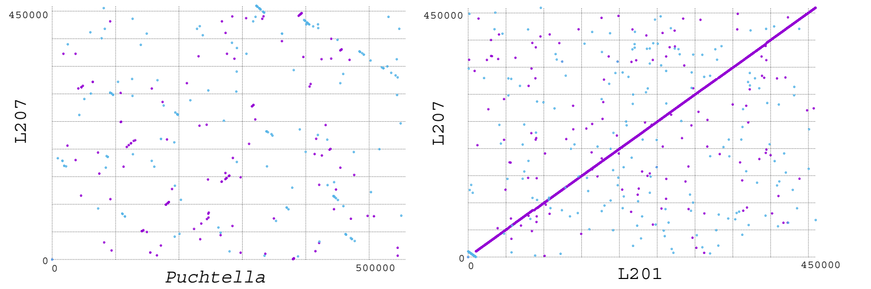

SupplementaryFigure4. Synteny of the five most complete genome drafts produced by Clinker (A) and Mauve (B). The contigs containing only 23S rRNA genes (see text) are not included. The colours in A show homology of the genes as recognized by Clinker. Grey arrows in B stand for the concatenated contigs, yellow arrows for individual genes (the genes partly overlapping with their neighbours are shown in the second row), red arrows show position of 16S rRNA gene. C - Average nucleotide identity (%) among the five *L. neohaematopini* strains and the closest relative *Puchtella* sp. str. PRUG (the table shows coverage of the genomes aligned for each pair comparison). D - similarities of 16S rRNA genes of the compared genome drafts. E - Dot plot comparison of *Puchtella* sp. str. PRUG and L207 genomes (left) in comparison with dotplot of L207 and L201 genomes (right). The direct and reversed matches are shown by purple and blue dots, respectively.

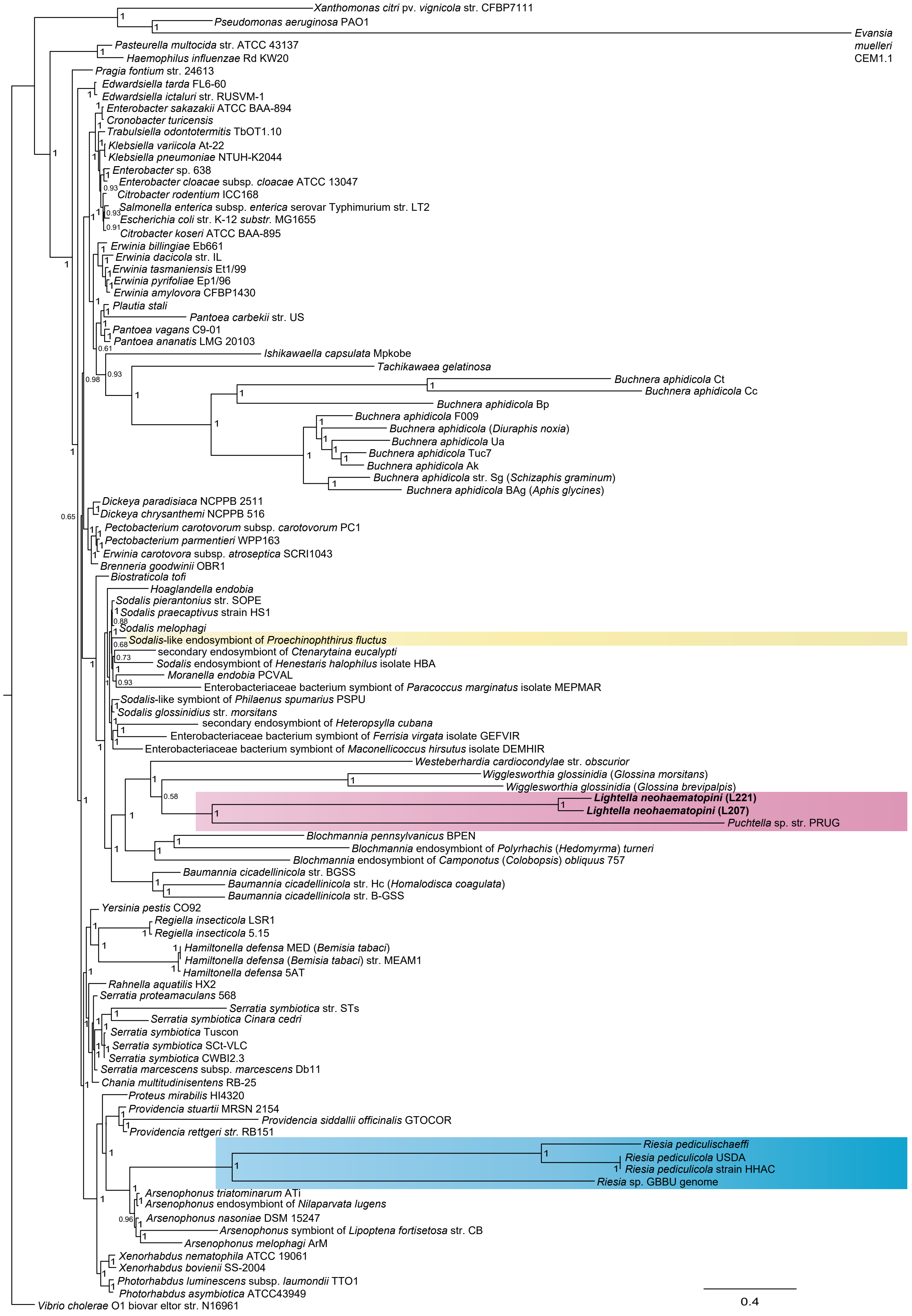

SupplementaryFigure5: Phylogenetic tree of Enterobacteriaceae (γ-proteobacteria) derived from the 14-protein “phylogenetic matrix” (7,438 aa) by PhyloBayes (BI under the CAT-GTR model). Clustering of *L. neohaemotopini* together with the *Puchtella* sp. str. PRUG is indicated by pink background. Other two lineages of lice symbionts originated within this bacterial order are highlighted by yellow and blue background. Values at the nodes of the tree show posterior probabilities.

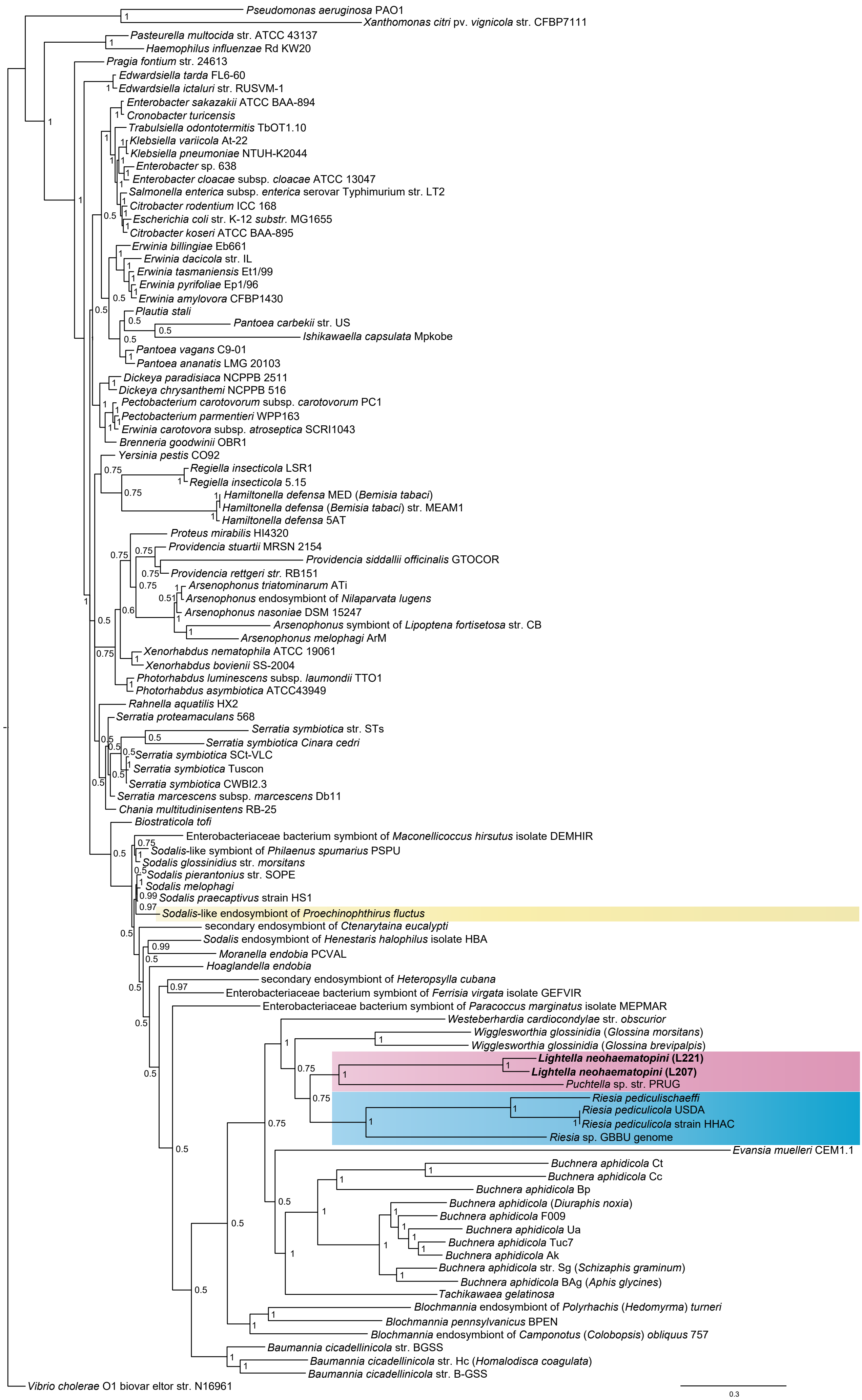

SupplementaryFigure6: Phylogenetic tree of Enterobacteriaceae (γ-proteobacteria) derived from the 14-protein "phylogenetic matrix" (7,438 aa) by MrBayes (BI using CpREV+G+I model). Clustering of *L. neohaemotopini* together with the *Puchtella* sp. str. PRUG is indicated by pink background. Values at the nodes of the tree show posterior probabilities.

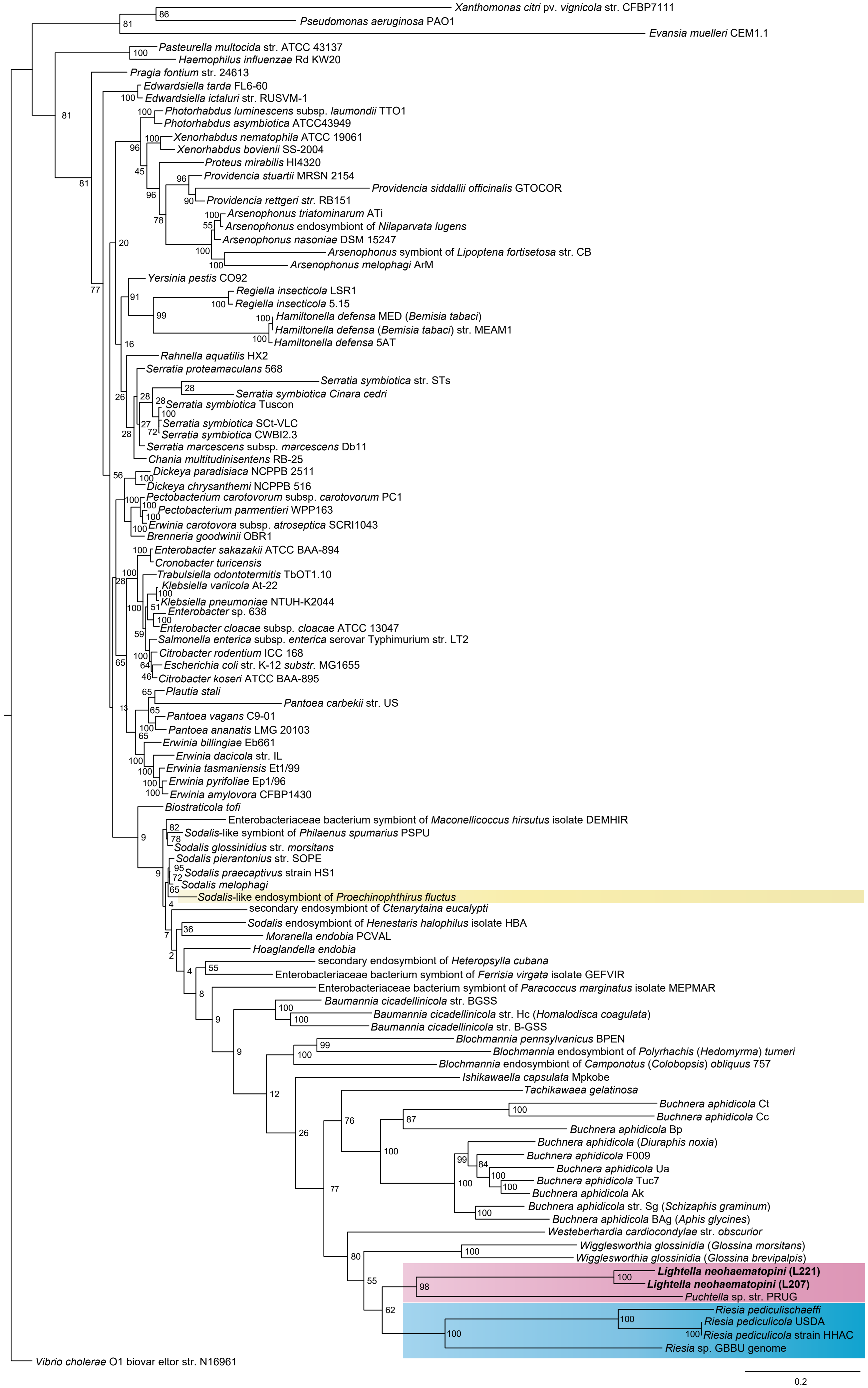

SupplementaryFigure7: Phylogenetic tree of Enterobacteriaceae (γ-proteobacteria) derived from the 14-protein "phylogenetic matrix" (7,438 aa) by PhyML (ML using CpREV+G+I model). Clustering of *L. neohaemotopini* together with the *Puchtella* sp. str. PRUG is indicated by pink background. Values at the nodes of the tree show bootstrap supports.

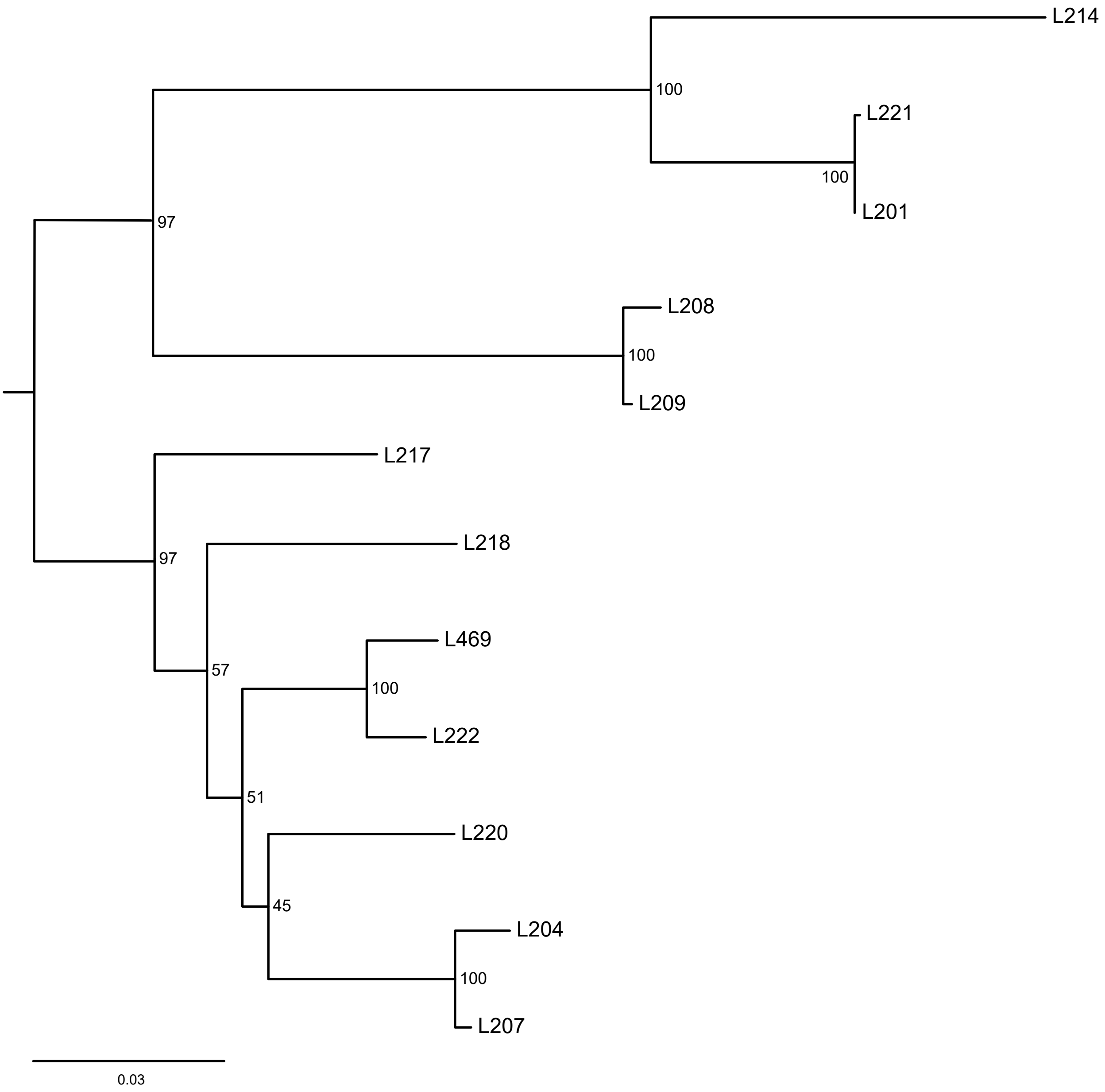

SupplementaryFigure8: Phylogenetic reconstruction of 5-protein "coevolutionary matrix" (1,150 aa) of *Lightella neohaematopini* samples inferred by PhyML (ML using HIVb+G+F model). Values at the nodes of the tree show posterior probabilities.

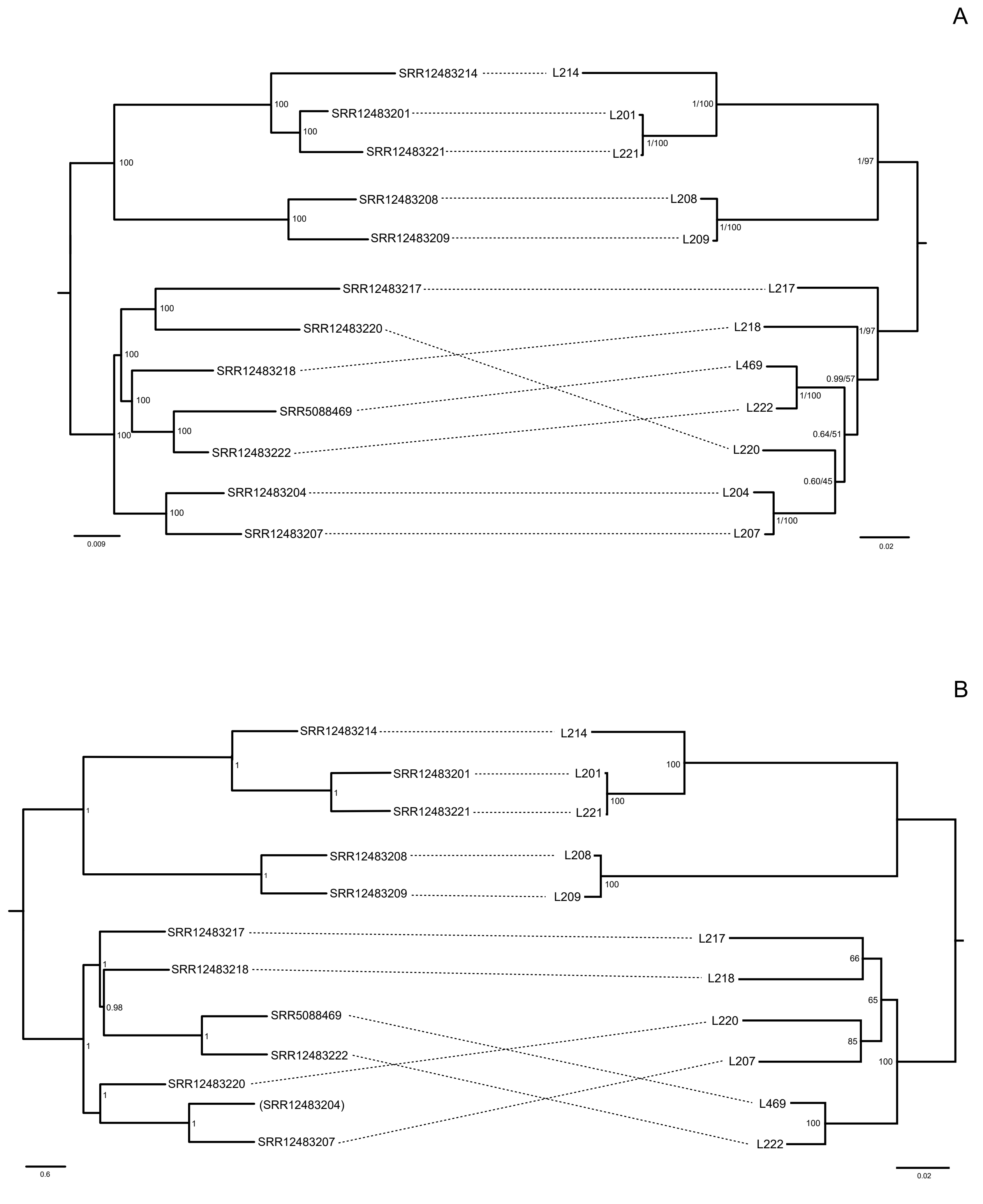

SupplementaryFigure9: A: Coevolutionary reconstruction comparing phylogenetic trees of the lice inferred by IQ-TREE (ML using GTR+F+R10 model) from 1,107 nuclear nucleotide loci and *L. neohaematopini* inferred by MrBayes (BI using JTT+G+F model) and PhyML (ML using HIVb+G+F model) from the amino-acid five-gene “coevolutionary matrix“ (1,150 aa). Lengths of the branches in the symbiont tree correspond to the BI results. Values at the nodes of the symbiont tree show posterior probabilities/ bootstrap supports. B: Coevolutionary reconstruction comparing phylogenetic trees of the lice inferred by ASTRAL-III method from 1,107 individual gene trees, each tree derived from one of the 1,107 nucleotide matrices by IQ-TREE using GTR+F+R10 model (this tree corresponds to the one presented in Figure 2) and the symbiont tree with reduced set of 11 samples (L204 removed) inferred by PhyML (ML using Q.bird+G+F model) from amino-acid fifty-gene matrix. The sample SRR12483204 shown in parenthesis corresponds to the removed L204 symbiont. Values at the nodes of the symbiont tree show bootstrap supports. Values at the nodes of the host tree show local posterior probabilities.

Pathway	Gene	*L. neohaematopini* L207	*Puchtela* sp. str. *PRUG*	*Neisseriaceae symbiont N206	*Neisseriaceae symbiont PsAf
Fatty acid biosynthesis	fabK	■	■		■
	fabF			■	■
	fabG	■	■	■	■
	fabZ	■	■		■
	fabI	■	■		■
	fabD	■	■	■	■
	fabH	■	■		
Purine metabolism	prsA	■	■		■
	purB		■	■	■
	purC			■	■
	purD			■	■
	purF			■	■
	purK			■	■
	purN			■	■
	purQ			■	■
	purSL			■	■
	purM			■	■
	purE			■	■
	purA			■	■
	purH			■	■
	gmk	■	■	■	■
	dgt			■	■
	surE				■
	nrdA	■	■	■	■
	nrdB	■	■	■	■
	guaA	■	■	■	■
	guaB		■	■	■
Pyrimidine metabolism	carB			■	■
	pyrB			■	■
	pyrC			■	■
	pyrD		■	■	■
	pyrE			■	■
	pyrF			■	■
	pyrH	■	■	■	■
	pyrG		■	■	■
	surE				■
	ndk	■		■	■
	dut	■	■	■	■
	tmk	■	■	■	■
	thyX/thyA			■	■
	carA			■	■
Heme metabolism	ALAS			■	■
Porphyrin and chlorophyll metabolism	gltX	■	■		■
	hemB			■	■
	hemC			■	■
	hemD			■	■
	hemE			■	■
	hemF/hemN				■
	hemJ/hemG			■	■
	ctaB	■			
	ctaA				
	bfr			■	■
Oxidative phosphorylation	RIP1				
	CYTB				
	CYC1				
	COX10	■			
	COX11				
	COX15				
	CoxC				
	CoxA				
	CoxB				
	Cyt. bd ubiquinol oxidase - cydA		■	■	■
	Cyt. bd ubiquinol oxidase - cydB		■	■	■
Secretion system	Type I - tolC				
	Type II - gspD				
	Sec-SRP - secA			■	■
	Sec-SRP - secB			■	■
	Sec-SRP - secD			■	■

		L. neohaematopini L207	*Puchtela* sp. str. *PRUG*	*Neisseriaceae symbiont N206	*Neisseriaceae symbiont PsAf
	Sec-SRP - secF				
	Sec-SRP - secG				
	Sec-SRP - secY				
	Sec-SRP - YajC				
	Sec-SRP - YidC				
	Sec-SRP - ftsY				
	Sec-SRP - ffh				
	secE				
	Twin arginine targeting - TatA				
	Twin arginine targeting - TatC				
	Type IV - virB10				
	virB3				
	virB4				
	virB9				
	virB6				
	virB8				
	virB11				
	virD4				
**Cell cycle**	murG				
	rseP				
	lon				
	dnaB				
	dnaA				
	PerP				
	cell division - ftsW				
	cell division - ftsZ				
	cell division - ftsQ				
	cell division - ftsA				
	two-component - pleC				
	two-component - pleD				
	ATP-dependent Clp - clpX				
	ATP-dependent Clp - clpP				
**Recombination**	ssb				
	polA				

		L. neohaematopini L207	*Puchtela* sp. str. *PRUG*	*Neisseriaceae symbiont N206	*Neisseriaceae symbiont PsAf
	dnaE				
	recJ				
	recA				
	recF				
	recO				
	recR				
	recG				
	ruvA				
	ruvB				
	ruvC				
**ABC transporters**	heme exporter - CcmA				
	heme exporter - CcmB				
	heme exporter - CcmC				
	heme exporter - CcmD				
	phosphate trasport system - PstA				
	phosphate trasport system - PstB				
	phosphate trasport system - PstC				
	phosphate trasport system - PstS				
	lipoprotein releasing system - lolC_E				
	lipoprotein releasing system - lolD				
	zinc transport system - ZnuA				
	zinc transport system - ZnuB				
	zinc transport system - ZnuC				
	biotin trasport system - BioY				
	biotin trasport system - EcfT				
	biotin trasport system - EcfA1				
	biotin trasport system - EcfA2				
	phospholipid trasport system – MlaC-D				
	phospholipid trasport system – MlaE-B				
	phospholipid trasport system - MlaF				

		L. neohaematopini L207	Puchtela sp. str. PRUG	*Neisseriaceae symbiont N206	*Neisseriaceae symbiont PsAf
	lipopolysaccharide - LptF				
	lipopolysaccharide - LptG				
	lipopolysaccharide - LptB				
Glycerophospholipid metabolism	gpsA				
	plsY				
	plsC				
	pssA				
	psd				
	cdsA				
	pgpA				
	pgsA				
	araM				
	dgkA				
	pldA				
Asparagine, aspartate	ansA				
Arginine	argA				
	argB				
	argC				
	argD				
	argG				
	argF (argI, OTC)				
	argJ				
	argH				
Phenylalanine	pheA				
	aspC				
Tryptophan	trpD				
	trpG				
	trpC				
	trpA				
	trpF				
	trpB				
Sulphate, cysteine	cysN				
	cysD				
	cysC				

		L. neohaematopini L207	Puchtela sp. str. PRUG	*Neisseriaceae symbiont N206	*Neisseriaceae symbiont PsAf
	cysH				
	cysI				
	cysJ				
	cysQ				
	cysE				
	cysK				
Methionine	metA				
	metB				
	metC				
	metE				
Lysine	asd				
	dapA				
	dapB				
	dapD				
	dapE				
	dapF				
	lysA				
	thrA				
	2.6.1.17 - argD				
Threonine	thrB				
	thrC				
Leucine	leuA				
	leuC				
	leuD				
	leuB				
Glycine	glyA				
Histidine	hisA				
	hisB				
	hisC				
	hisD				
	hisF				
	hisG				
	hisH				
	hisI				

		L. neohaematopini L207	*Puchtela* sp. str. *PRUG*	*Neisseriaceae symbiont N206	*Neisseriaceae symbiont PsAf
**Serine**	**serC**				
**Chorismate**	**aroG**				
	**aroB**				
	**aroD**				

		L. neohaematopini L207	*Puchtela* sp. str. *PRUG*	*Neisseriaceae symbiont N206	*Neisseriaceae symbiont PsAf
	**aroE**				
	**aroK**				
	**aroA**				
	**aroC**				

SupplementaryFigure10: Comparison of the main metabolic pathways for *L. neohaematopini* with its closest relative *Puchtella* sp. str. PRUG (γ-proteobacteia in blue background) and Neisseriaceae-related symbiont N206 with its closest relative Neisseriaceae-related symbiont PsAf (β-proteobacteria in yellow background). Presence of the genes is indicated by grey background. *Neisseriaceae-related.

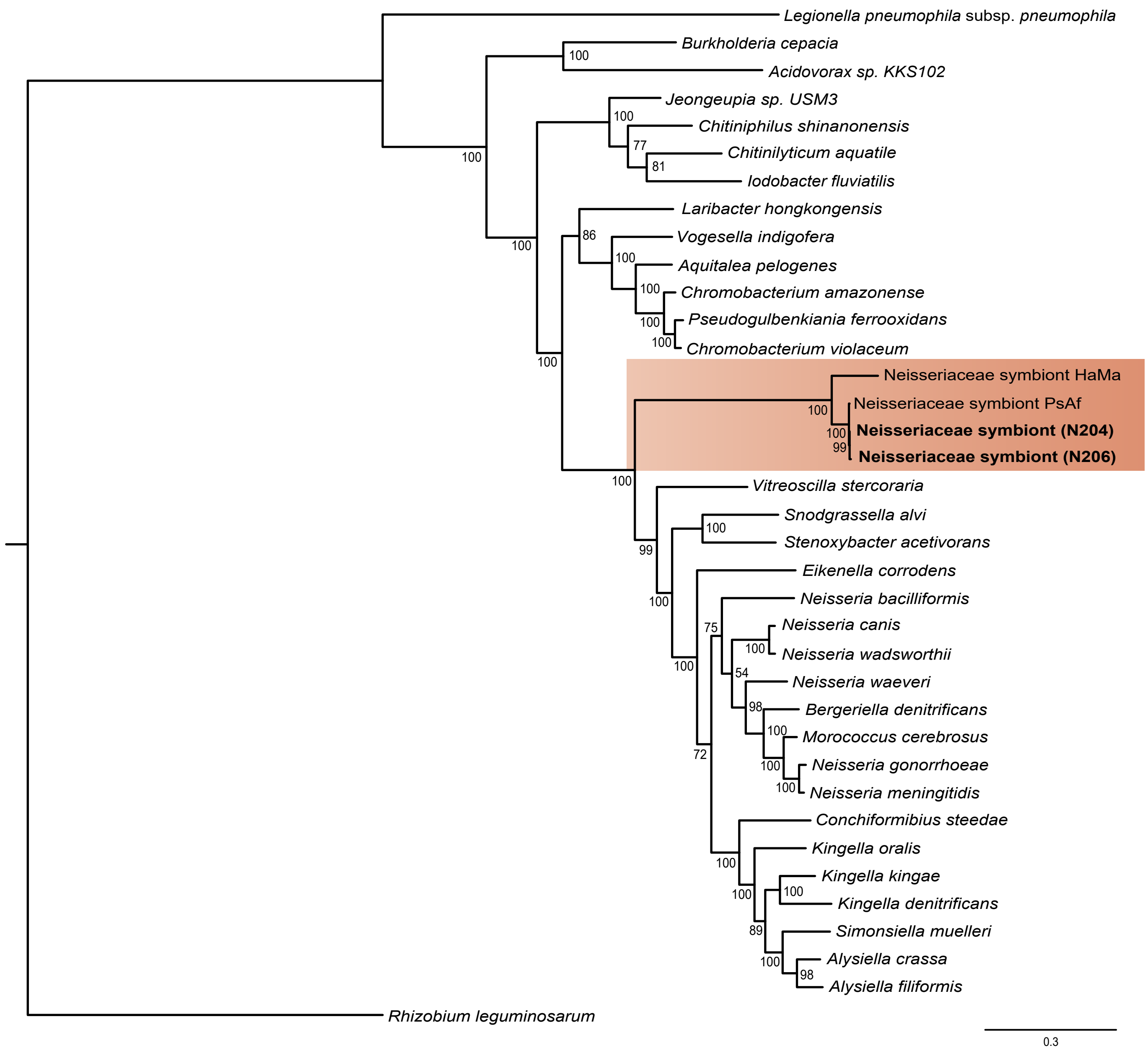

SupplementaryFigure11: Phylogenetic reconstruction of the amino-acid 30-gene “Neisseriales matrix” (4,465 aa) of Neisseriales obtained with PhyML (ML using LG+G+I+F model). Values at the nodes of the tree show bootstrap supports.
